# Supplementary material for: A previously undescribed archaeal virus suppresses host immunity
Source: EMBO Rep. 2025 Nov 17;26(24):6159–78. doi: 10.1038/s44319-025-00540-3 (PMC12714723; doi:10.1038/s44319-025-00540-3)
Supplement: Supplementary file 1 — Appendix [file 44319_2025_540_MOESM1_ESM.pdf]

Table of contents – Appendix file

Item	Page
Appendix Table S1 -	2
Appendix Table S2	3
Appendix Table S3	4
Appendix Table S4	5
Appendix Figure S1	6
Appendix Figure S2	7
Appendix Figure S3	8
References for the appendix	9

Appendix Table S1: Numbers of spacers acquired by *H. volcanii* CRISPR-Cas after mating with *Haloferax* strain Atlit 48N (5 biological replicates)

A.	Number of unique spacer matches							
	48N chromosome	pWL-102	*H. volcanii* chromosome	phv1	phv3	phv4	p48N_2	Multiple hits
Replicate 1	47	5	18	2	8	6	1	78
Replicate 2	178	18	44	11	27	26	4	206
Replicate 3	201	14	25	1	25	10	2	128
Replicate 4	276	30	56	5	42	21	2	247
Replicate 5	107	5	34	1	13	14	4	92

B.	Total number of spacer matches							
	48N chromosome	pWL-102	*H. volcanii* chromosome	phv1	phv3	phv4	p48N_2	Multiple hits
Replicate 1	406	5	18	2	8	6	1	82
Replicate 2	6067	18	45	11	27	26	4	229
Replicate 3	7303	14	26	1	26	10	2	157
Replicate 4	6110	32	58	5	45	21	2	542
Replicate 5	1119	8	36	1	13	14	5	250

Appendix Table S2 - Summary of the spacer acqusition experiment conducted in 48N and its virus-cured derivative. Numbers indicate independent biological replicates.

Sample	depth	Filtered candidates	Spacers passing BLAST threshold	Spacers with bad matches	Total spacers	Duplcates (using spacer BLAST criteria)	Mutated duplicates	Total duplicates	Spacer to duplicate ratio	Mutated duplicate fraction
cured-A-1	12963435	23280	95	6	101	2.15E+04	1.45E+02	2.17E+04	4.66E-03	6.70E-03
cured-A-2	19743770	114501	5054	129	5183	1.01E+05	7.08E+02	1.01E+05	5.11E-02	6.99E-03
cured-A-3	12468171	56242	301	12	313	4.72E+04	5.12E+02	4.77E+04	6.57E-03	1.07E-02
wt-A-1	7631842	22363	310	8	318	2.03E+04	1.56E+02	2.04E+04	1.56E-02	7.63E-03
wt-A-2	4875663	10396	96	4	100	9.47E+03	7.20E+01	9.54E+03	1.05E-02	7.55E-03
wt-A-3	6133899	15882	123	3	126	1.42E+04	1.29E+02	1.43E+04	8.79E-03	9.00E-03
cured-B-1	2413316	52360	210	22	232	4.21E+04	2.59E+03	4.47E+04	5.19E-03	5.80E-02
cured-B-2	2383356	47769	145	12	157	4.15E+04	2.06E+03	4.36E+04	3.60E-03	4.73E-02
cured-B-3	3571418	75847	389	38	427	6.35E+04	4.07E+03	6.76E+04	6.32E-03	6.02E-02
wt-B-1	2696743	119036	128	50	178	1.00E+05	6.86E+03	1.07E+05	1.66E-03	6.39E-02
wt-B-2	2860390	274506	802	113	915	2.42E+05	1.22E+04	2.54E+05	3.60E-03	4.79E-02
wt-B-3	2014598	119813	287	51	338	1.05E+05	5.46E+03	1.11E+05	3.05E-03	4.93E-02
cured-C-1	5720783	186301	83	14	97	1.68E+05	2.28E+03	1.70E+05	5.70E-04	1.34E-02
cured-C-2	4899258	31897	147	4	151	3.01E+04	3.27E+02	3.05E+04	4.96E-03	1.07E-02
cured-C-3	6138998	154958	400	12	412	1.47E+05	2.06E+03	1.49E+05	2.77E-03	1.38E-02
wt-C-1	5679309	403317	1011	51	1062	3.44E+05	4.60E+03	3.49E+05	3.05E-03	1.32E-02
wt-C-2	5160729	153132	632	25	657	1.09E+05	1.55E+03	1.11E+05	5.92E-03	1.40E-02
wt-C-3	5412240	191639	263	15	278	1.78E+05	2.20E+03	1.81E+05	1.54E-03	1.22E-02

Mutated duplicates - duplicate spacers that had fewer than 10 mismatches to spacer in the original array but did not meet BLASTN thresholds

Appendix Table S3: Oligonucleotides used in this study

Primer	sequence (5'-3')	Comments
IT748	CGGGTTCGGACTCGCGCTCGG	genotype confirmation for the 48N *ura*⁻ strain, forward primer
IT749	CGGGGCGTTCGAGGTCCAGCG	genotype confirmation for the 48N *ura*⁻ strain, reverse primer
IT5	CACCGAGGACGAACTCGAA	HLSV1 upstream forward primer, for generating 48N HLSV1 Δintegrase
IT6	CCGATTCAGCCTTAATGGGTATGGGACCGCCCGGATTTGAACCGGGGTCACG	HLSV1 upstream reverse primer, for generating 48N HLSV1 Δintegrase
IT7	TCAAATCCGGGCGGTCCCATACCCATTAAGGCTGAATCGGCCGTTCTTGC	HLSV1 downstream forward primer, for generating 48N HLSV1 Δintegrase
IT8	CACCGAGTGAGTTCGGGC	HLSV1 downstream reverse primer, for generating 48N HLSV1 Δintegrase
IT1	CACCGAGGACGAACTCGAA	HLSV1 upstream forward primer, for generating 48N ΔHLSV1
IT2	CCGATTCAGCCTTAATGGGTATGGGACCGCCCGGATTTGAACCGGGGTCACG	HLSV1 upstream reverse primer, for generating 48N ΔHLSV1
IT3	TCAAATCCGGGCGGTCCCATACCCATTAAGGCTGAATCGGCCGTTCTTGC	HLSV1 downstream forward primer, for generating 48N ΔHLSV1
IT4	CACCGAGTGAGTTCGGGC	HLSV1 downstream reverse primer, for generating 48N ΔHLSV1
IT24	CCTCCTCGAAGCGATAACAG	virus 48N ddPCR Fw-Amplify 48N virus for ddPCR
IT25	CGAGTTTCTCTCGGGTGTTC	virus 48N ddPCR Rv-Amplify 48N virus for ddPCR
IT556	CGCGGGAACGACTTTCGAC	HLSV1 integrated form -down forward 1000bp
IT557	CCGTCCAGTGGGACGTTATC	HLSV1 integrated form -down reverse 1000bp
IT558	AATCAGATGAACGTCGCCCT	HLSV1 integrated form -up forward 1000bp
IT559	CGACGATGCCGTGTGTCTAC	HLSV1 integrated form -up reverse 1000bp
IT560	GTCGATCACGTGGCATCAG	HLSV1 forward circular - 500bp
IT561	ACGAAGGGGAGGTCCGTC	HLSV1 reverse circular - 500bp
IT270	GGGTCGACGGAAACGTTGAT	Forward- used for new CRISPR spacers acquisitions in CRISPR array C and D of *Hfx. volcanii* lab strain
IT271	AATTGGACCCCGGCTTCG	Reverse- used for new CRISPR spacers acquisitions in CRISPR array D of *Hfx. volcanii* lab strain
IT272	TGTGATTCGATACGCGACAC	Reverse- used for new CRISPR spacers CRISPR acquisitions in CRISPR array C of *Hfx. volcanii* lab strain
IT548	CCGTACTCAGACCACGACA	Forward- used for new CRISPR spacers acquisitions in CRISPR array A of 48N
IT549	CGTAGTCACCCCTCAGAGAGT	Reverse- used for new CRISPR spacers acquisitions in CRISPR array A of 48N
IT550	GACAATTCGCTCGGTCACG	Forward- used for new CRISPR spacers acquisitions in CRISPR array B of 48N
IT551	TGATTTCGGGACGGTTTCAG	Reverse- used for new CRISPR spacers acquisitions in CRISPR array B of 48N
IT552	GGCTTCGACGGGGATTGTC	Forward- used for new CRISPR spacers acquisitions in CRISPR array C of 48N
IT553	GGGTCGACGGAAACACTCTT	Reverse- used for new CRISPR spacers acquisitions in CRISPR array C of 48N

Appendix Table S4: plasmids used in this study

Plasmid	Description	Source / Reference
pGB68	containing the halobacterial novobiocin resistance gene *gyrB* and flanking sequences of *pyrE2*	(Bitan-Banin *et al*, 2003)
pIS71	Pop in-pop out plasmid used to delete the LSV-48N integrase gene (stock reference name UG587)	This study
pWL102	*Escherichia coli-Haloferax volcanii* shuttle vector. confers resistance to ampicillin and mevinolin	(Lam & Doolittle, 1989)

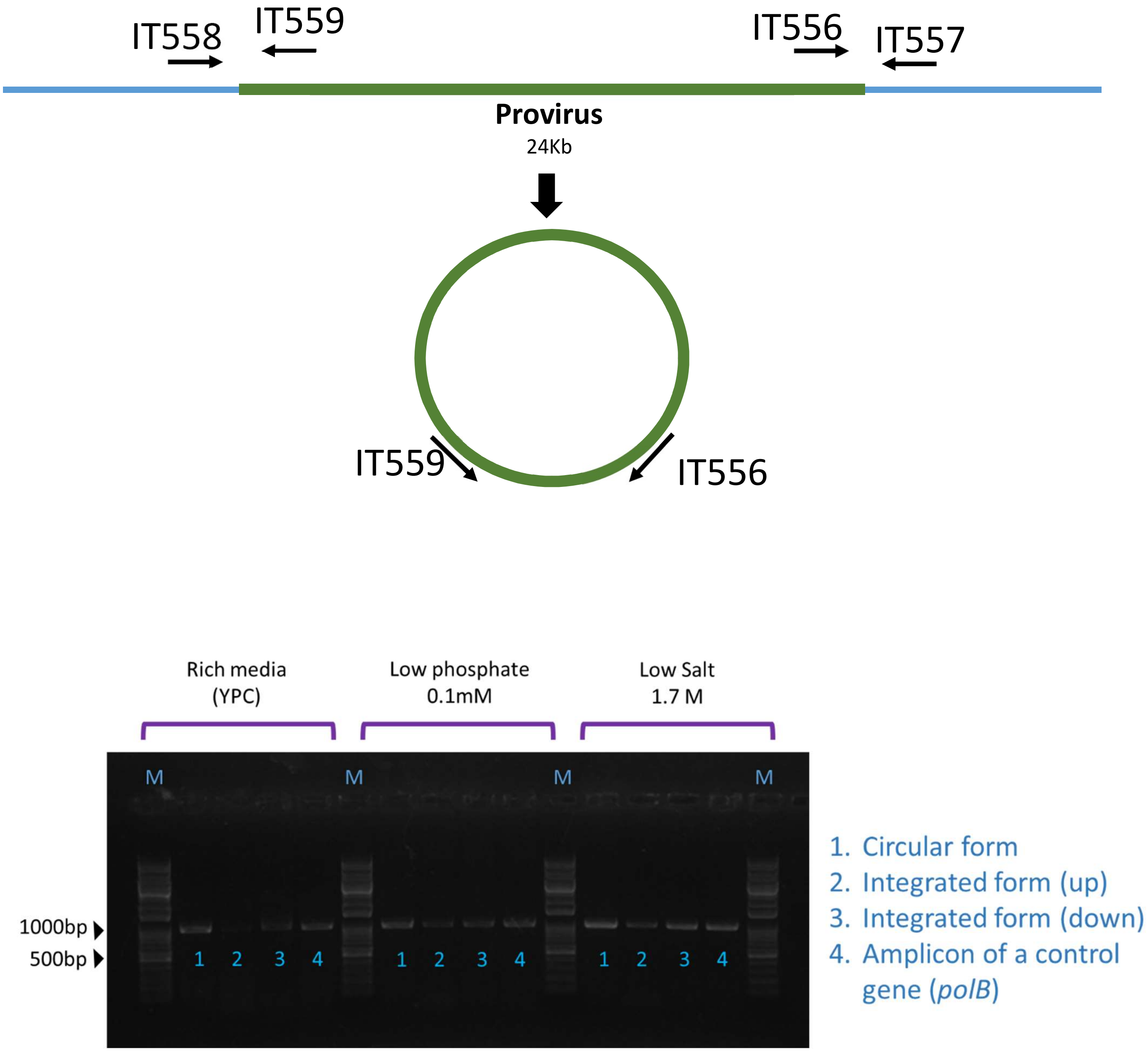

Appendix Figure S1. Evidence for both replicating and integrated forms of LSV-48N in cells. Agarose gel electrophoresis of PCR amplicons obtained from liquid cultures of 48N cells grown under different conditions.

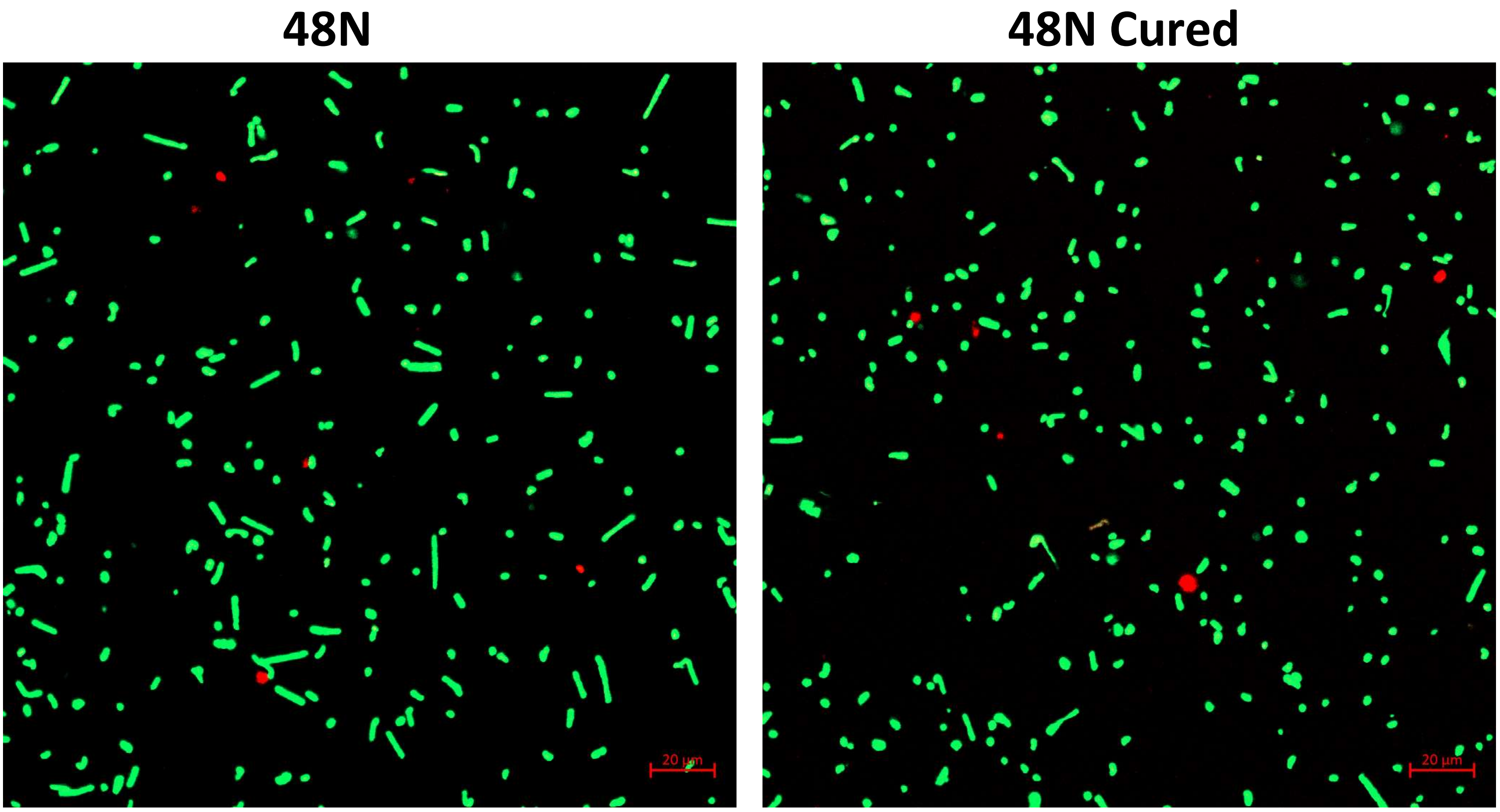

Appendix Figure S2. Live-dead staining of 48N WT and 48N-cured strains, visualized by fluorescent microscopy

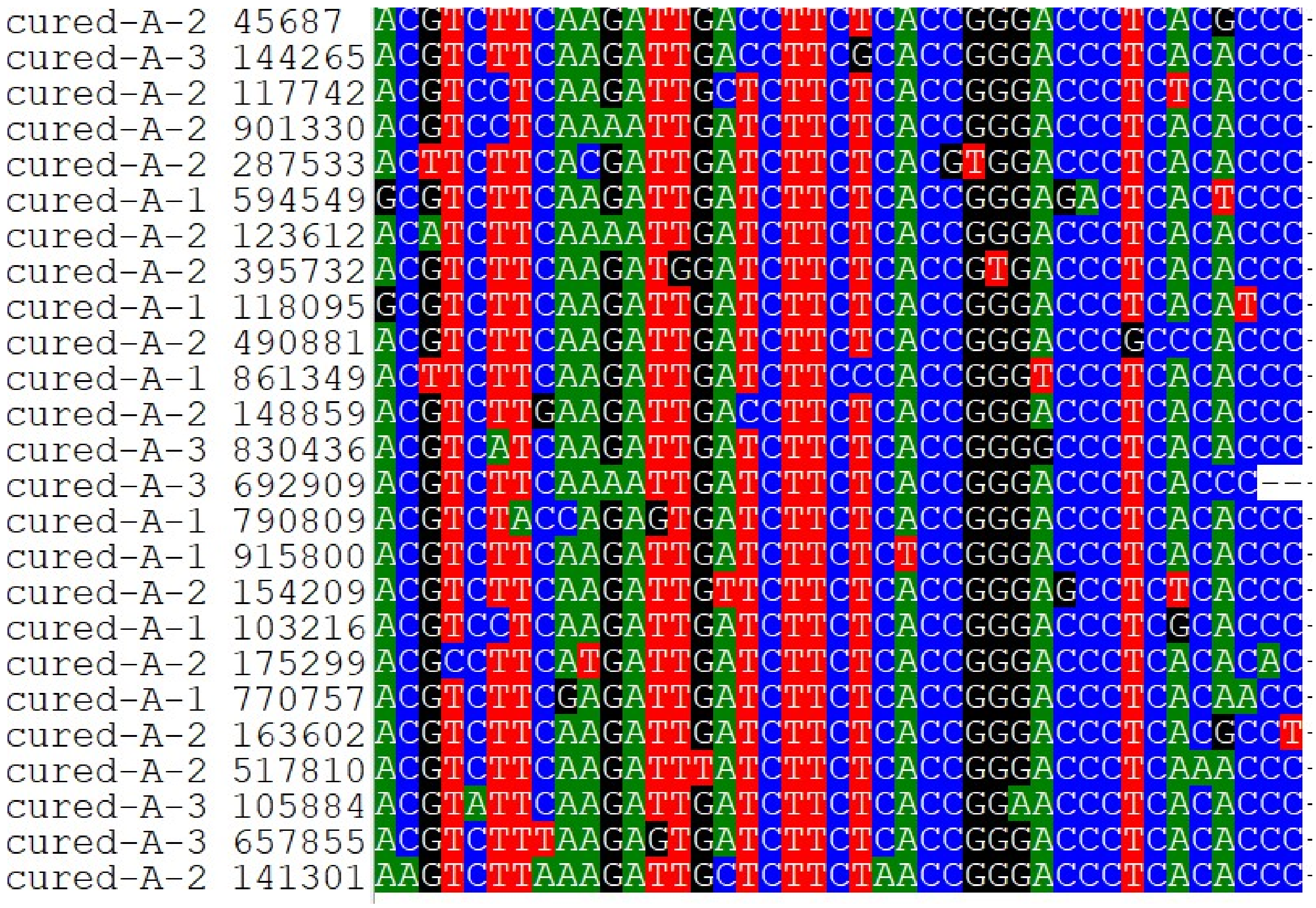

Appendix Figure S3. Mutated duplicated spacers observed in spacer acquisition assays of 48N cells. In this case representative sequences from the virus-cured 48N are shown.

References for the Appendix

Bitan-Banin G, Ortenberg R, Mevarech M (2003) Development of a gene knockout system for the halophilic archaeon Haloferax volcanii by use of the pyrE gene. *Journal of bacteriology* 185: 772-778

Lam WL, Doolittle WF (1989) Shuttle vectors for the archaebacterium Halobacterium volcanii. *Proceedings of the National Academy of Sciences of the United States of America* 86: 5478-5482
