# Supplementary material for: A previously undescribed archaeal virus suppresses host immunity
Source: EMBO Rep. 2025 Nov 17;26(24):6159–78. doi: 10.1038/s44319-025-00540-3 (PMC12714723; doi:10.1038/s44319-025-00540-3)
Supplement: Supplementary file 9 — Expanded View Figures [file 44319_2025_540_MOESM9_ESM.pdf]

Expanded View Figures

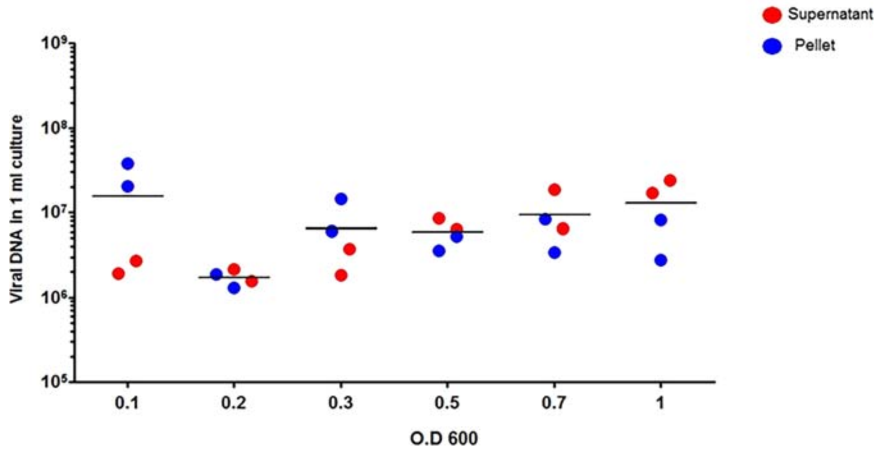

Figure EV1. Digital droplet PCR quantification of LSV-48N genome copies along the growth curve (increasing OD).

Taken from a culture of 48N grown on rich (YPC) medium. Two biological replicates are shown.

© The Author(s)

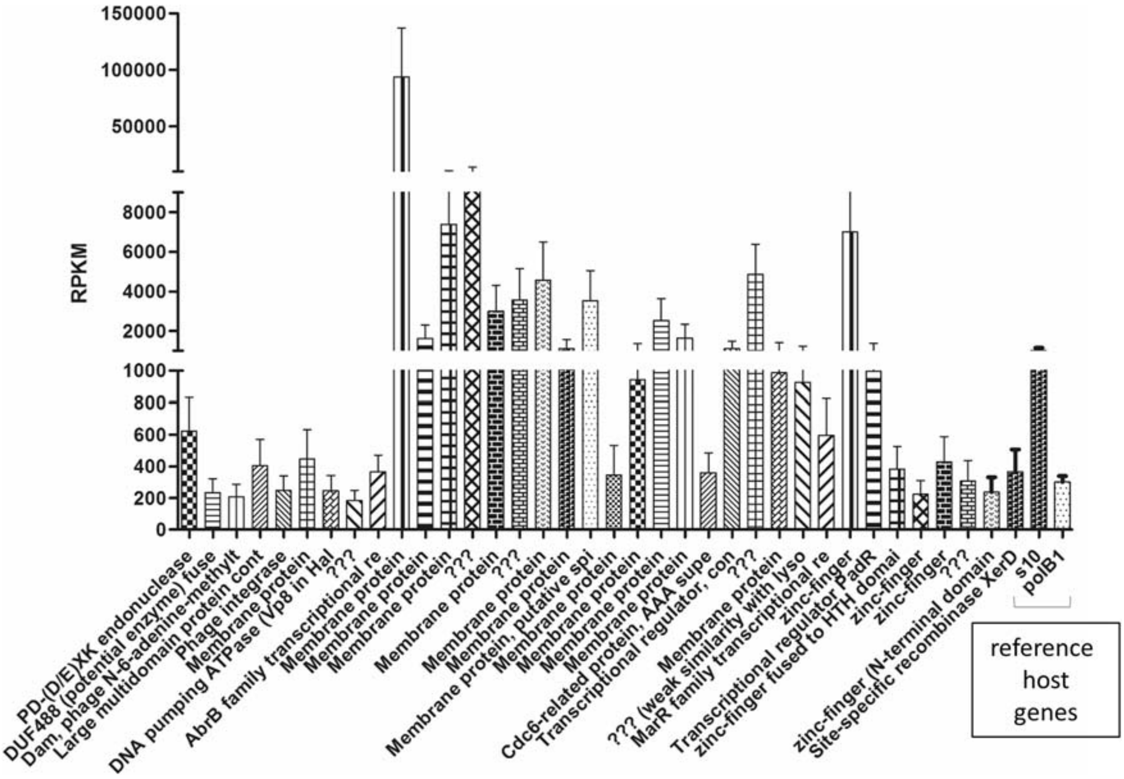

Figure EV2. Mean gene expression levels of the LSV-48N genes in a virus-infected 48N culture.

Based on three 3 RNA-Seq experiments (biological replicates). A highly-expressed host gene and a moderately-expressed host gene are provided as a reference. Genes of unknown function are marked as "???".

 © The Author(s)

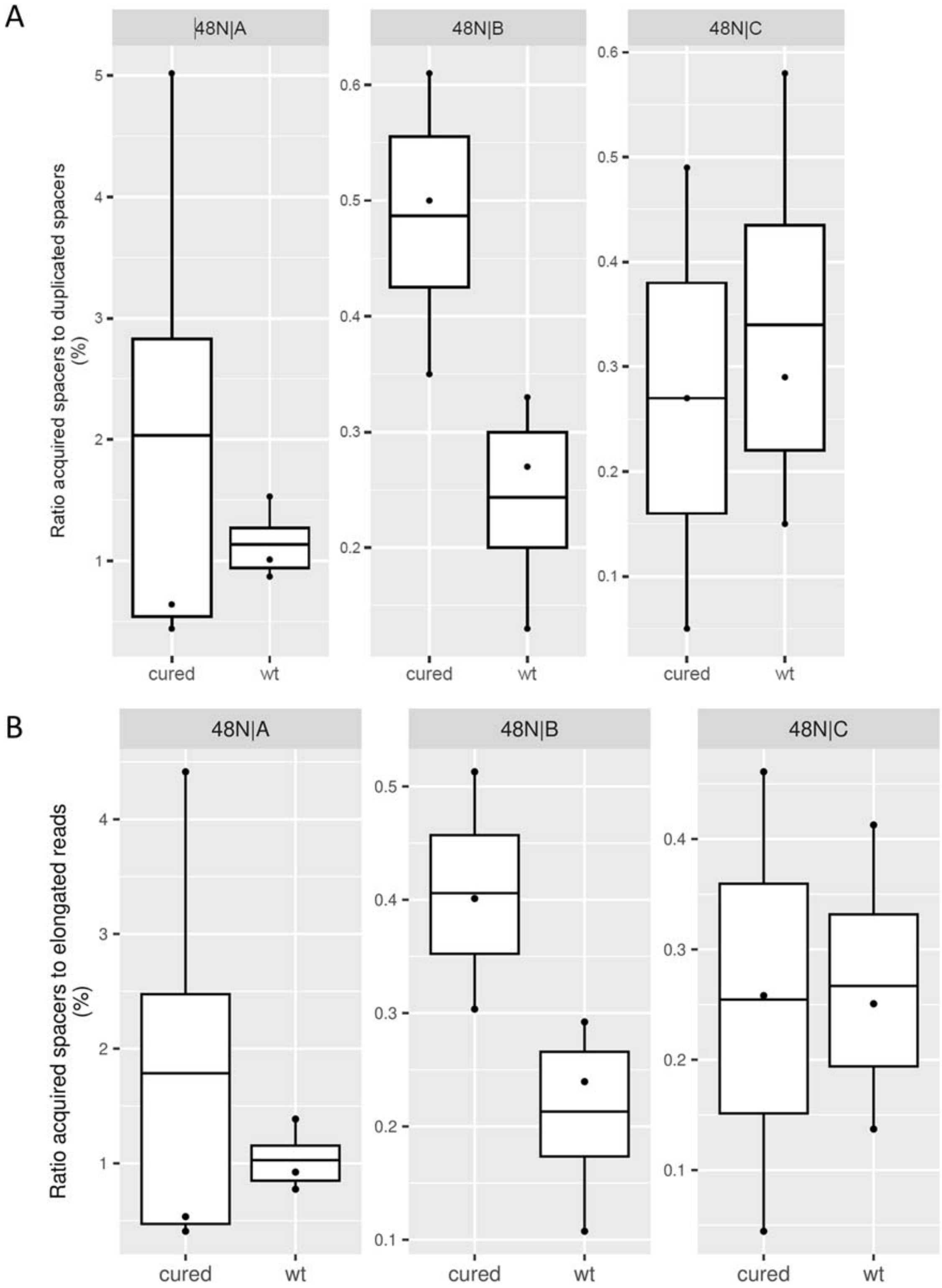

Figure EV3. Spacer acquisition in the three CRISPR arrays, A, B and C, in 48N and its virus-cured derivative.

Lines within bars are the mean of the three biological replicates, shown as dots. (**A**) The ratio of acquired spacers vs. duplicated spacers. (**B**) Ratio of acquired spacers vs. all reads of length corresponding to the length of an extended array, indicating a potential spacer acquisition event.

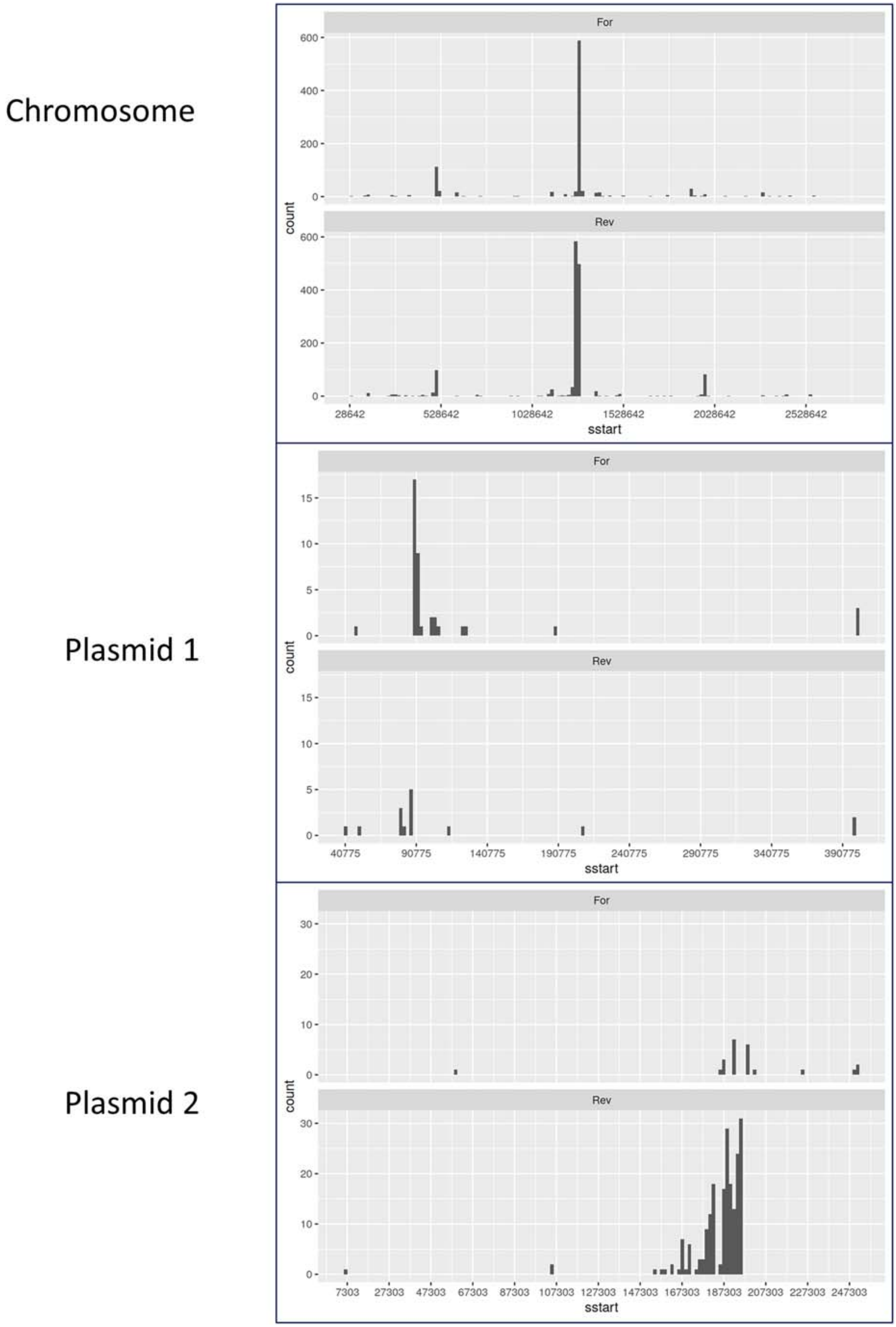
Chromosome
For
Rev
count
sstart
28642
528642
1028642
1528642
2028642
2528642
Plasmid 1
40775
90775
140775
190775
240775
290775
340775
390775
Plasmid 2
7303
27303
47303
67303
87303
107303
127303
147303
167303
187303
207303
227303
247303

 © The Author(s)

◀ **Figure EV4. Spacer acquisition event count by genomic location.**

Data include three biological replicates, over all three arrays in both strains.

© The Author(s)

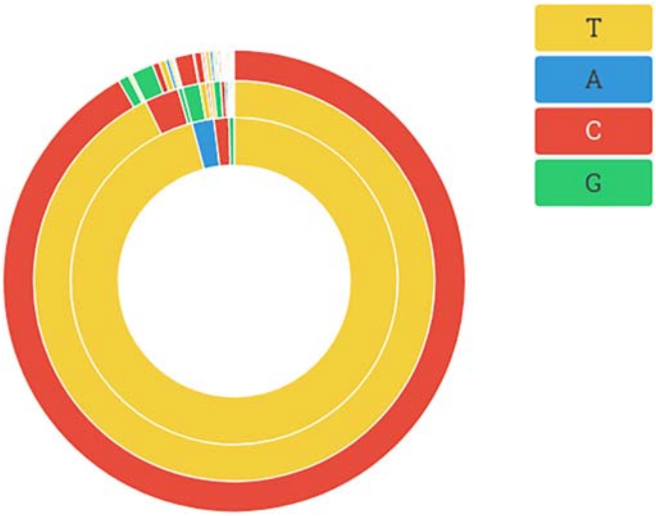

Figure EV5. PAM wheel (Leenay et al, 2016) representation of protospacer adjacent motifs in the spacers acquired by 48N.

Inner circle corresponds to the spacer-proximal nucleotide. Data include all new spacer acquisition events from the three biological replicates.
